# Discrimination of Falsified Erectile Dysfunction Medicines by Use of an Ultra-Compact Raman Scattering Spectrometer

**DOI:** 10.3390/pharmacy9010003

**Published:** 2020-12-24

**Authors:** Tomoko Sanada, Naoko Yoshida, Kazuko Kimura, Hirohito Tsuboi

**Affiliations:** 1 Clinical Pharmacy and Healthcare Sciences, Faculty of Pharmacy, Institute of Medical, Pharmaceutical and Health Sciences, Kanazawa University, Kakuma-Machi, Kanazawa 920-1192, Ishikawa, Japan; tmk810@stu.kanazawa-u.ac.jp (T.S.); tsuboih@p.kanazawa-u.ac.jp (H.T.); 2 AI Hospital/Macro Signal Dynamics Research and Development Center, Institute of Medical, Pharmaceutical and Health Sciences, Kanazawa University, Kakuma-Machi, Kanazawa 920-1192, Ishikawa, Japan; 3 Medi-Quality Security Institute, Graduate School of Medical Sciences, Kanazawa University, Kakuma-Machi, Kanazawa 920-1192, Ishikawa, Japan; kimurak@p.kanazawa-u.ac.jp

**Keywords:** Cialis, Levitra, Viagra, ultra-compact Raman scattering spectrometer, handheld, principal component analysis, identification

## Abstract

Substandard and falsified medicines are often reported worldwide. An accurate and rapid detection method for falsified medicines is needed to prevent human health hazards. Raman scattering spectroscopy has emerged as a non-destructive analysis method for the detection of falsified medicines. In this laboratory study, Raman spectroscopy was performed to evaluate the applicability of the ultra-compact Raman scattering spectrometer (C13560). Principal component analysis (PCA) was also performed on the Raman spectra. This study analyzed tadalafil (Cialis), vardenafil (Levitra), and sildenafil (Viagra) tablets. We tested the standard product and products purchased from the internet (genuine or falsified). For Cialis and Levitra, all falsified tablets were identified by the Raman spectra and PCA score plot. For Viagra, the Raman spectra of some falsified tablets were almost comparable to the standard tablet. The PCA score plots of falsified tablets were dispersed, and some plots of falsified tablets were close to the standard tablet. In conclusion, C13560 was useful for the discrimination of falsified Cialis and Levitra tablets, whereas some falsified Viagra tablets had Raman spectra similar to that of the standard tablet. The development of detection methods that can be introduced in various settings may help prevent the spread of falsified products.

## 1. Introduction

Substandard and falsified medicines have been increasing worldwide and have become a global threat. “Falsified” medicines intentionally misrepresent the details of products, “substandard” medicines are authorized medical products that do not meet quality standards, and “unregistered/unlicensed” medicines have not been approved for sale under the relevant regulations and legislation, as per World Health Organization (WHO) definitions [1]. Substandard and falsified medicines put people’s health at risk [2]. The exact number of substandard and falsified medical products worldwide cannot be determined. However, according to an estimate published by the Center for Medicine in the Public Interest in the United States of America, substandard and falsified medicine sales exceeded 75 billion US dollars in 2010 [3]. The WHO Global Surveillance and Monitoring System for substandard and falsified medical products reported that up to 2017, there were 1500 reports of substandard and falsified medical products [4]. These products include not only pharmaceuticals, such as antibiotics, antidiabetic agents, and antimalarial drugs, but also lifestyle medicines for beauty, diet, and erectile dysfunction (ED) [4,5,6,7,8,9]. The WorldWide Antimalarial Resistance Network (WWARN) published the data of falsified medicines from across the world [10]. Especially in low- and middle-income countries (LMICs), it is estimated that one-tenth of medicines are substandard or falsified [2,11]. Poor-quality medicines cause patient harm, such as direct hazards to health and loss of trust in medical care. Furthermore, substandard and falsified medicines cause economic damage, such as lost earnings for genuine manufacturers and funding for criminal organizations. Therefore, it is necessary to take measures to prevent the distribution of substandard and falsified products.

The Asia Pacific Economic Cooperation indicated that it is important to develop methods and techniques to detect and prevent falsified products in their Roadmap for Supply Chain Security. However, the development of detection methods has been slow, and no reliable method has emerged [12,13]. Conventionally, high-performance liquid chromatography (HPLC) and thin-layer chromatography (TLC) have been used as pharmaceutical analysis techniques, and these analyses can accurately qualify and quantify active pharmaceutical ingredients (API). A handheld device has also been developed that can not only be used in the laboratory or a special facility but also in the field. Minilab^®^ developed by Global Pharma Health Fund e.V. (Giessen, Germany), which is a rapid analysis of TLC, has been used all over the world including in LMICs and evaluated for its usefulness [14,15,16]. However, HPLC and TLC are destructive analyses (consuming samples and failing to retain them), require machine operator training, and require the availability of reference standards [17]. Because these destructive analyses require time and a skilled operator, spectroscopic analysis has been widely used as an alternative method in recent years [18]. Spectroscopy is a non-destructive technique and requires no special training or pretreatment [17,19]. Spectroscopic analysis, including near-infrared and Raman spectroscopy, has been applied in various fields, such as food [20,21,22,23], plants [24], art [25], treatment [26,27], and medical products [28,29,30,31,32,33,34,35,36,37,38,39,40,41]. Raman scattering analysis is included in the United States Pharmacopoeia and the European Pharmacopoeia, and many major pharmaceutical companies worldwide use Raman spectroscopy to identify source materials [18].

Pharmaceutical Inspection Convention and Pharmaceutical Inspection Co-operation Scheme (PIC/S) are the co-operative agreements on Good Manufacturing Practices (GMP) for pharmaceuticals; the aim of these agreements is to establish a common standard for GMP and harmonize testing procedures. PIC/S indicates that the identity of the batch of starting materials is certified only if it is tested on each sample taken from all packages [42,43]. Therefore, an accurate and quick detection method for testing a large number of samples is required. Portable analyzers can be used in the field and reduce the number of samples that need to be tested in the laboratory [14,44,45]. Several handheld Raman scattering analyzers with different functions, sizes, and prices have been developed, and there are several reports that have evaluated their usefulness [7,8,14,44,45,46,47,48,49,50,51]. The handheld Raman spectrometer is expected to be used in customs, warehouses, medical institutions, and pharmacies, and is cheaper than fixed Raman spectrometers installed in laboratories. However, when used in low-income institutions or LMICs, it is crucial that the cost of the analyzer and the number of consumables required for each analysis is low [52]. Therefore, we evaluated the usefulness of the ultra-compact Raman scattering spectrometer (C13560, Hamamatsu Photonics K.K., Shizuoka, Japan) developed in Japan. Compared with the handheld Raman scattering spectrometers that have already been reported, the advantages of the ultra-compact Raman scattering spectrometer are its price and size. Its size is similar to a smartphone, and it weighs only 90 g. In addition to its small size, the cost of the ultra-compact Raman scattering spectrometer is low enough to be affordable in many places and is one-tenth the price of the analyzer that we used in previous studies [7,8]. However, it needs to be modified for application because it is an Original Equipment Manufacturing (OEM) Raman spectroscopic module.

In this study, we examined the usefulness of the ultra-compact Raman spectrometer as a non-destructive detection method. The method was applied to detect falsified ED medicines that were in distribution; the authentic products were: tadalafil (Cialis), vardenafil (Levitra), and sildenafil (Viagra) [7,8,9]. In addition to the confirmation of the Raman spectra, principal component analysis (PCA) was also performed to classify products.

## 2. Materials and Methods 

### 2.1. Materials

This study analyzed three medicines for ED, including Cialis (20 mg tablet), Levitra (20 mg tablet), and Viagra (100 mg tablet). Standard Cialis 20-mg and Levitra 20-mg tablets were obtained through Japanese legal distribution [53,54]. The Viagra 100-mg tablet is an unapproved dosage form in Japan, and thus standard Viagra 100-mg tablets were obtained from the manufacturer and distributor, Pfizer Inc. (New York, NY, USA) [55,56]. 

We tested products obtained by purchasing over the internet in previous studies [7,8,9]. These products were previously judged as genuine or falsified. Authenticity was assessed by the manufacturer of the product. The quality, such as the quantity of APIs and dissolution of the product, was confirmed in previous studies [7,8,9]. For Cialis tablets (n = 33), one standard product, nine genuine products (GC1– GC9, n = 9), and 23 falsified products (FC1–FC23, n = 23) were tested. For Levitra tablets (n = 23), one standard product, nine genuine products (GL1–GL9, n = 9), and 13 falsified products (FL1–FL13, n = 13) were tested. For Viagra tablets (n = 23), one standard product, four genuine products (GV1–GV4, n = 4), and 18 falsified products (FV1–FV18, n = 18) were tested.

The ingredients used as excipients in Cialis, Levitra, and Viagra tablets were obtained as much as possible [53,54,56]. Magnesium stearate was purchased from FUJIFILM Wako Pure Chemical Corporation (Osaka, Japan). Talc was purchased from Maruishi Pharmaceutical. Co., Ltd. (Osaka, Japan). Hydroxypropyl cellulose 150–400 cP and titanium oxide were purchased from Wako Pure Chemical Industries, Ltd. (Osaka, Japan). Hydroxypropyl methylcellulose was purchased from Alfa Aesar, Thermo Fisher Scientific (Lancashire, United Kingdom). Lactose was purchased from KENEI Pharmaceutical Co., Ltd. (Osaka, Japan). A reference standard for tadalafil and sildenafil citrate was purchased from the United States Pharmacopeia (Rockville, MD, USA). A reference standard for vardenafil dihydrochloride was purchased from LKT Laboratories, Inc. (St. Paul, MN, USA).

### 2.2. Raman Spectroscopy

**Figure 1:**
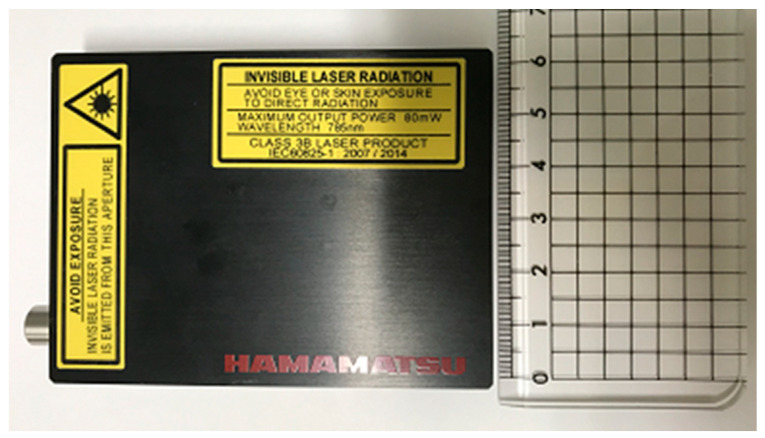
Image of the ultra-compact scattering spectrometer (C13560).

The Raman spectra of the tablet surface were measured using the ultra-compact scattering spectrometer (C13560, Figure 1). The size of the device was 96 mm × 14.5 mm × 60 mm, and the weight was 90 g. The laser excitation wavelength was 785 nm, the power was 15 mW, and the exposure time was one second. The spectral range of the detection area was 400–1850 cm^−1^. We used the focus guide provided by Hamamatsu Photonics to adjust the focus of the laser under the same conditions each time. As a preparation for the measurement, the dark was measured using a silicon substrate to subtract the dark data from the measurement data. The silicon peak detected near 520 cm^−1^ was used for calibration [57]. We tested five tablets of the standard product and one tablet of the genuine or falsified product. The surface of the tablet was measured five times, and the average of the intensity at each wavenumber of spectra was used to analyze the Raman spectra. The non-curved area of the tablet was irradiated with the laser, avoiding the manufacturer’s imprint. All additive agents and reference standards were placed in a clear plastic bag and measured five times from the outside of the package.

### 2.3. Multivariate Analysis

PCA was performed using The Unscrambler X 10.5 (CAMO Software, Oslo, Norway). As the pre-processing step, a Gaussian function filter method with a segment size set to 15 for smoothing, baseline correction, and normalizing with the maximum value was performed on all averaged spectra. In the PCA score plot, n-dimensional spectral data were converted into two-dimensional data and shown as plots in the Figure. PCA score plots and loading plots were used for grouping the products and to confirm spectral peaks for identification.

## 3. Results

### 3.1. Analysis of Components

**Figure 2:**
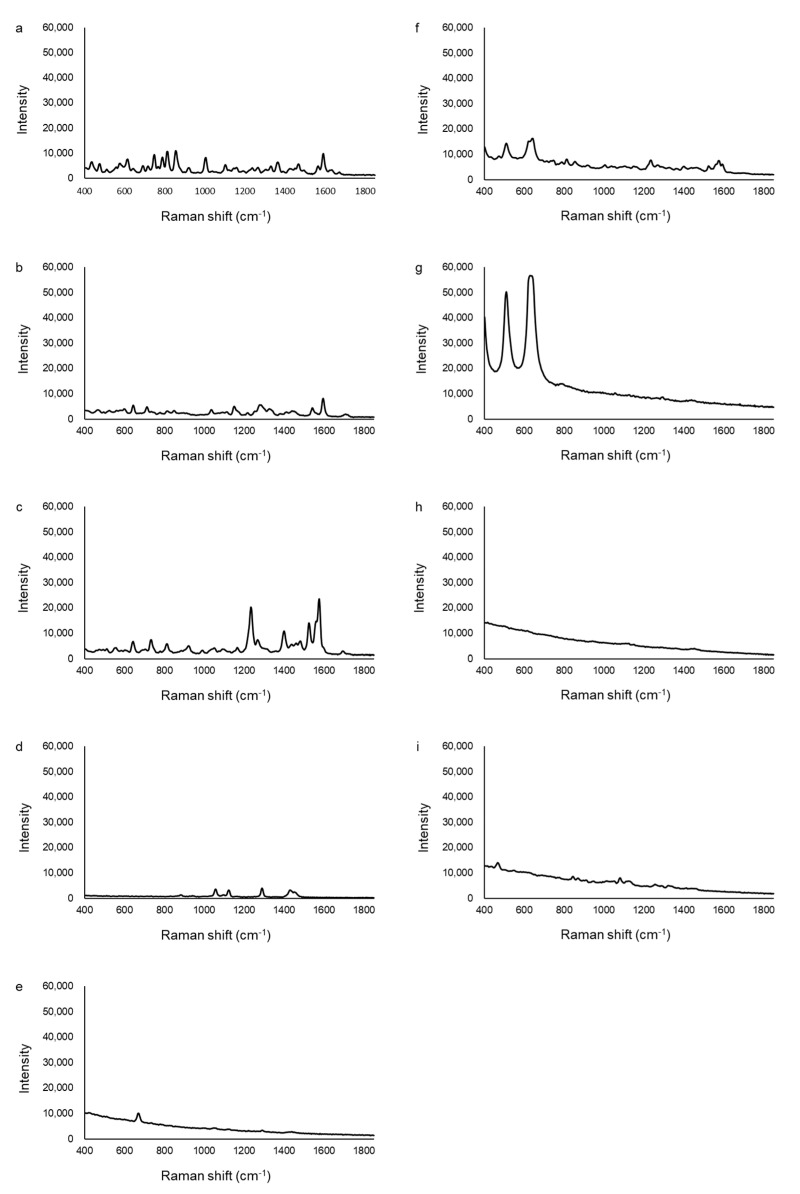
The Raman spectra of additive agents and reference standards. (**a**) tadalafil, (**b**) vardenafil dihydrochloride, (**c**) sildenafil citrate, (**d**) magnesium stearate, (**e**) talc, (**f**) hydroxypropyl cellulose, (**g**) titanium oxide, (**h**) hydroxypropyl methylcellulose, (**i**) lactose.

The Raman spectra of additive agents and reference standards are shown in Figure 2. The reference standard for tadalafil, vardenafil dihydrochloride, and sildenafil citrate had some peaks between 400–1700 cm^−1^, and the common point of the three spectra was the peak around 1580–1600 cm^−1^ (Figure 2a–c). Sildenafil citrate had two large peaks with an intensity higher than 20000 around 1240 and 1580 cm^−1^ (Figure 2c). Magnesium stearate had small peaks around 1060, 1120, 1290, and 1430 cm^−1^ (Figure 2d). Talc had only one peak, which was around 670 cm^−1^ (Figure 2e). Hydroxypropyl cellulose and titanium oxide had two major peaks around 510 and 630 cm^−1^, but the intensity of the titanium oxide peak was significantly higher than that of hydroxypropyl cellulose (Figure 2f,g). Hydroxypropyl cellulose was contained in the Cialis tablet only, and titanium oxide was used in the Cialis, Levitra, and Viagra tablets [53,54,56]. There were no peaks in the spectra of hydroxypropyl methylcellulose, and the intensity decreased as the wavenumber increased (Figure 2h). Lactose had small peaks around 470, 840, and 1080 cm^−1^ (Figure 2i). 

### 3.2. Analysis of Cialis

**Figure 3:**
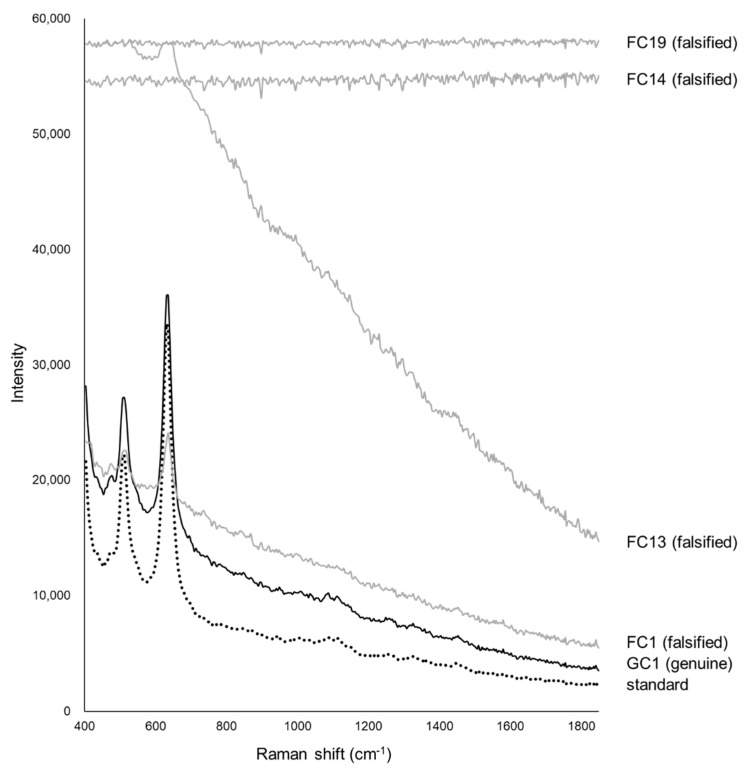
The Raman spectra of Cialis tablets. The black dotted line shows the spectra of the standard tablet (the average data of five standard tablets). The black lines show one of the genuine tablets (GC1), and the gray lines show four falsified tablets (FC1, FC13, FC14, FC19).

Figure 3 shows the Raman spectra of the standard, one genuine, and four falsified Cialis tablets. The two major peaks of the standard and genuine tablets (GC1–GC9) were obtained around 510 and 630 cm^−1^. The intensity of the peak around 630 cm^−1^ appeared to be more than twice as high as the peak around 510 cm^−1^ from the spectra baseline. Twelve falsified tablets (FC1–FC12) showed peaks at the same wavenumbers as the standard tablet but with differences in intensity. The intensity of the peak around 630 cm^−1^ was higher, but not twice as high (as in the standard tablet), than the peak around 510 cm^−1^ in the falsified tablets (FC1–FC12). One falsified tablet (FC13) had almost no peaks, and the intensity of the spectra decreased as the wavenumber increased. In the other 10 falsified tablets (FC14–FC23), no peaks were detected because there was fluorescence, and the Raman spectra were flat. Tablets with no peaks could not be distinguished visually by color differences. A curve toward the x-axis around 550–600 cm^−1^ was found in standard and genuine tablets, but the spectra were horizontal in all falsified tablets (FC1–FC23).

**Figure 4:**
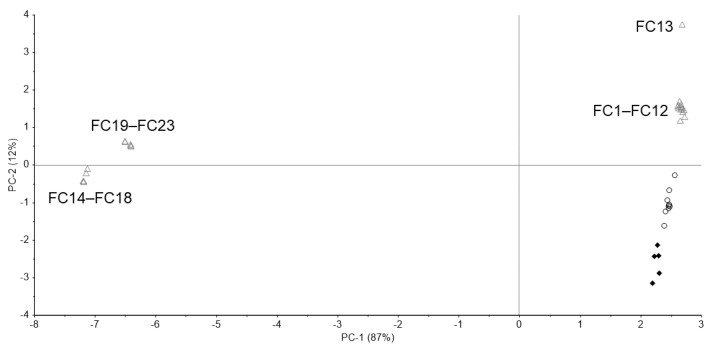
The principal component analysis (PCA) score plot of the Raman spectra of Cialis tablets. Black diamond; standard tablet (n = 5), black open circle; genuine tablets (n = 9), gray open triangle; falsified tablets (n = 23).

**Figure 5:**
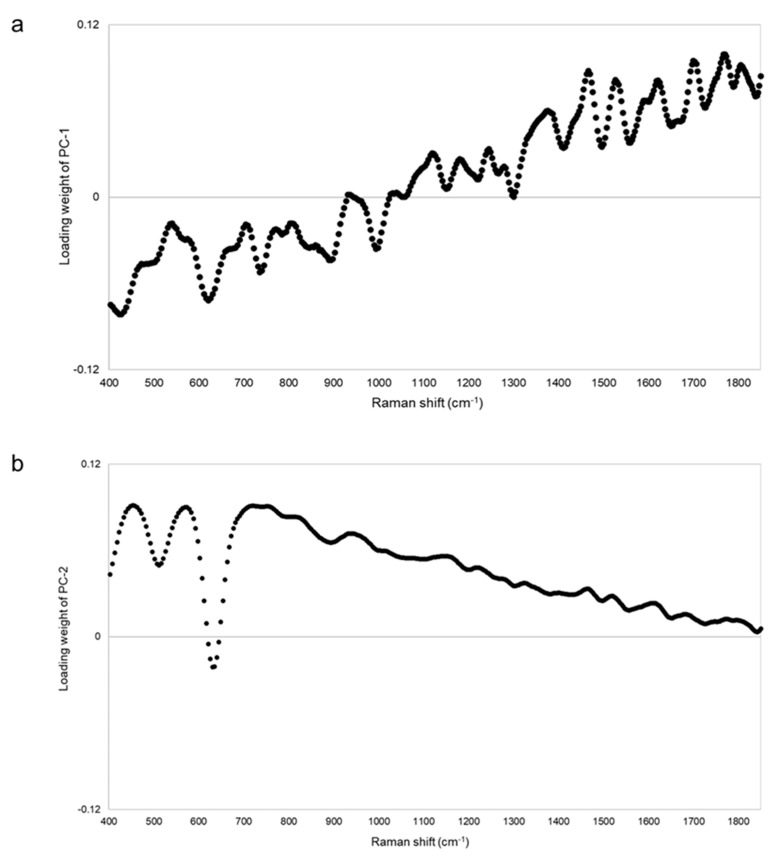
The PCA loading plot of the Raman spectra of Cialis tablets. Panels (**a**,**b**) show the loading plot of PC-1 and PC-2 of the Raman spectra of Cialis tablets, respectively.

In the PCA, the principal component (PC)-1 and PC-2 explained about 99% (PC-1 = 87%, PC-2 = 12%) of the spectra (Figure 4). Because genuine tablets were very close to standard tablets, standard and genuine tablets were considered to be similar. Ten falsified tablets (FC14–FC23) were significantly different from standard tablets in PC-1. Thirteen falsified tablets (FC1–FC13) were close to the standard tablet in PC-1 but different in PC-2. It was not possible to identify falsified tablets by PC-1 alone. From the loading plot, PC-1 was affected by the intensity of the peaks around 430, 625, and 1460–1850 cm^−1^ (Figure 5a). PC-2 was affected by the intensity of the peaks around 450, 570, 690–780 cm^−1^ (Figure 5b).

### 3.3. Analysis of Levitra 

**Figure 6:**
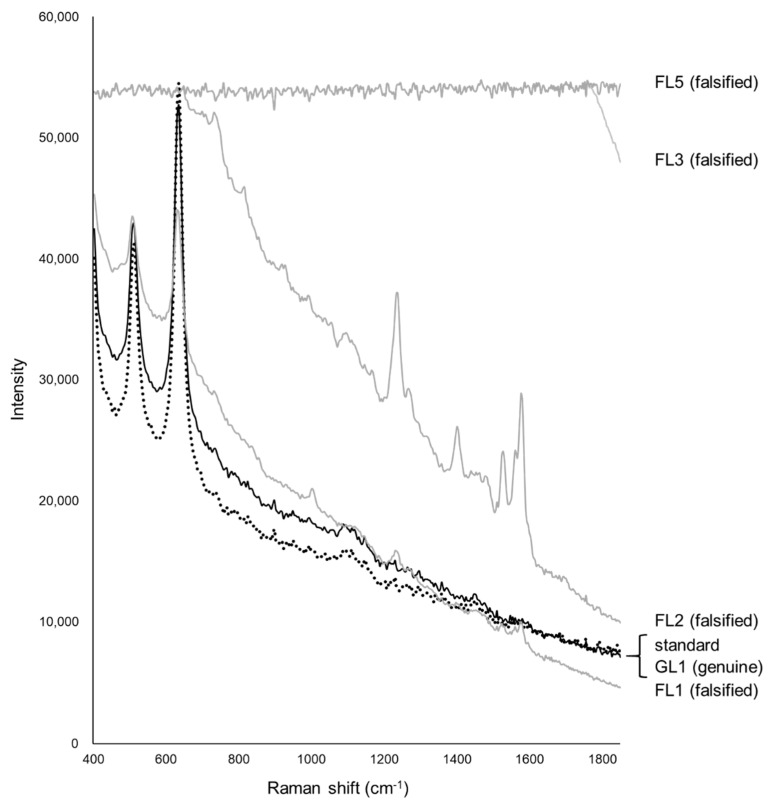
The Raman spectra of Levitra tablets. The black dotted line shows the spectra of the standard tablet (the average data of five standard tablets). The black lines show one of the genuine tablets (GL1), and the gray lines show four falsified tablets (FL1, FL2, FL3, FL5).

Figure 6 shows the Raman spectra of the standard, one genuine, and four falsified Levitra tablets. The two major peaks of the standard and genuine tablets (GL1–GL9) were obtained around 510 and 630 cm^−1^. One falsified tablet (FL1) also showed peaks around 510 and 630 cm^−1^ but at a different intensity compared with that of the standard tablet. Another falsified tablet (FL2) showed peaks around 1240, 1400, and 1520–1590 cm^−1^, which were quite different from the standard tablet. FL1 and FL2 showed spectra similar to those of sildenafil, and the major peak was clearly visible in FL2 (Figure 2 and Figure 6), suggesting that FL1 and FL2 are falsified tablets containing sildenafil. FL2 was visually different in color from the other falsified tablets and contained the highest amount of sildenafil among the falsified Levitra tablets obtained in a previous study [8]. In 11 falsified tablets (FL3–FL13), no peak was detected because there was fluorescence, and the spectra were flat. Tablets with no peaks could not be distinguished visually by color differences.

**Figure 7:**
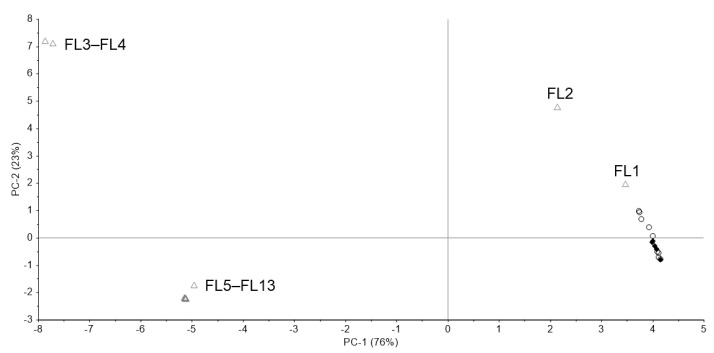
The PCA score plot of the Raman spectra of Levitra tablets. Black diamond; standard tablet (n = 5), black open circle; genuine tablets (n = 9), gray open triangle; falsified tablets (n = 13).

**Figure 8:**
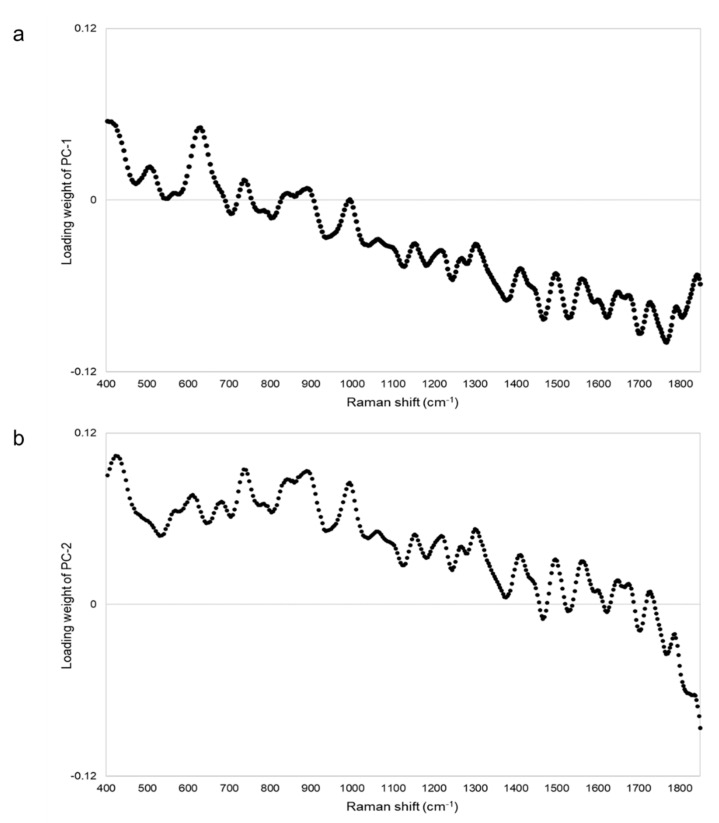
The PCA loading plot of the Raman spectra of Levitra tablets. Panels (**a**,**b**) show the loading plot of PC-1 and PC-2 of the Raman spectra of Levitra tablets, respectively.

Figure 7 shows the PCA score plot. PC-1 and PC-2 explained about 99% (PC-1 = 76%, PC-2 = 23%) of the spectra. Because the standard tablet was inside the group of genuine tablets, we could not distinguish between the standard and genuine tablets. One falsified tablet (FL1), which showed peaks around 510 and 630 cm^−1^ (Figure 6), was close to the group containing standard and genuine tablets. Another falsified tablet (FL2), which had different spectra compared with the standard tablet (Figure 6), was distinguished in both PC-1 and PC-2. Eleven falsified tablets (FL3–FL13) were significantly different from the standard tablet in PC-1. PC-2 was also assessed because the contribution of PC-1 was 76%, but almost all falsified tablets, except falsified tablet FL1, could be found using PC-1 alone. From the loading plot, PC-1 was affected by the intensity of the peaks around 410, 630, and 1460–1810 cm^−1^ (Figure 8a). PC-2 was affected by the intensity of the peaks around 420, 740, 870–900, 990, and 1850 cm^−1^ (Figure 8b). Although the loading plots of PC-1 and PC-2 appeared similar, the absolute value of the loading weight of PC-1 was high after 1450 cm^−1^, and the absolute value of the loading weight of PC-2 was high before 1000 cm^−1^.

### 3.4. Analysis of Viagra

**Figure 9:**
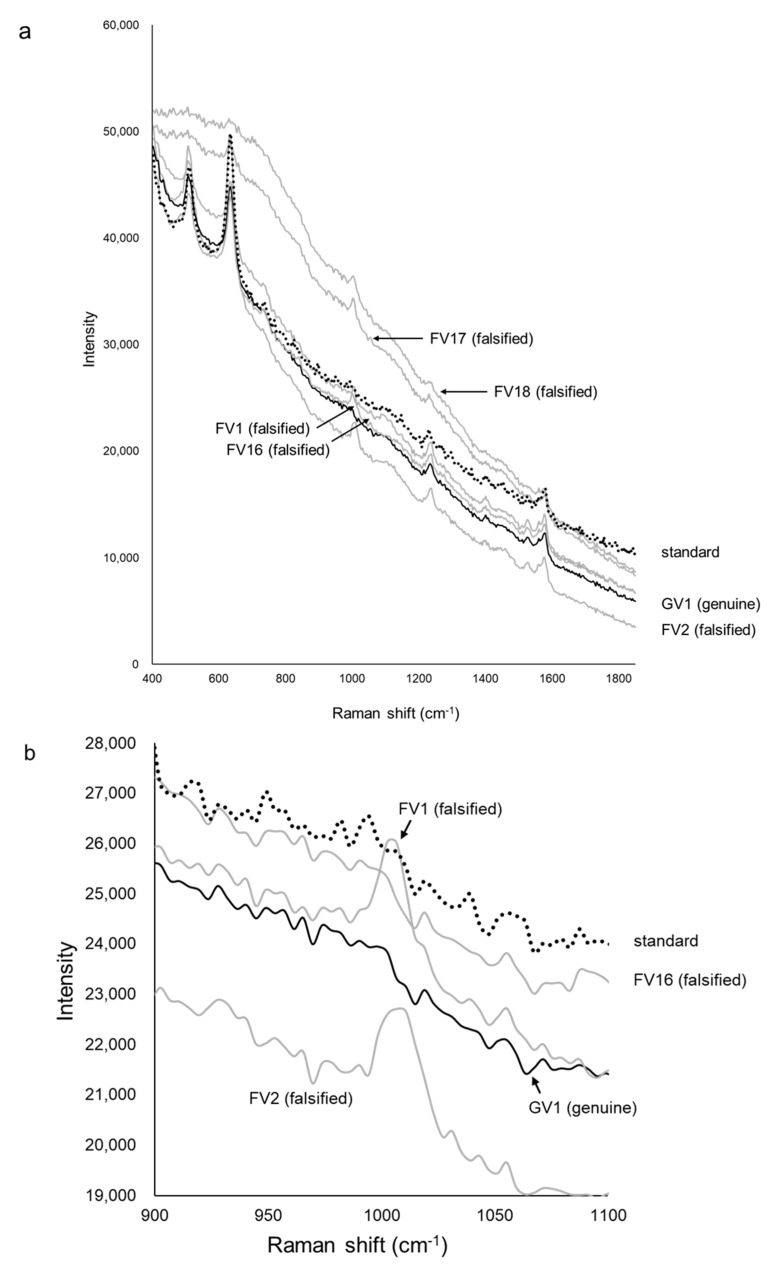
The Raman spectra of Viagra tablets. Panel (**a**) shows the Raman spectra of Viagra tablets. The black dotted line shows the spectra of the standard tablet (the average data of five standard tablets). The black lines show one of the genuine tablets (GV1), and the gray lines show five falsified tablets (FV1, FV2, FV16, FV17, FV18). Panel (**b**) is an enlargement of the area around 1000 cm^−1^ of panel a).

Figure 9a shows the Raman spectra of the standard, one genuine, and five falsified Viagra tablets. The two major peaks of the standard, genuine (GV1–GV4) and some falsified tablets were obtained around 510 and 630 cm^−1^. In addition, 17 of 18 falsified tablets (FV1–FV15, FV17, and FV18) showed a small peak around 1000 cm^−1^ that was not found in the standard and genuine tablets, but one falsified tablet (FV16) did not show this peak (Figure 9b). Two falsified tablets (FV17, FV18) had no major peaks, and the intensity of spectra decreased as the wavenumber increased. Because the spectra of some falsified tablets (FV1–FV16) were similar to those of the standard tablet, it was difficult to visually identify all of the falsified tablets.

**Figure 10:**
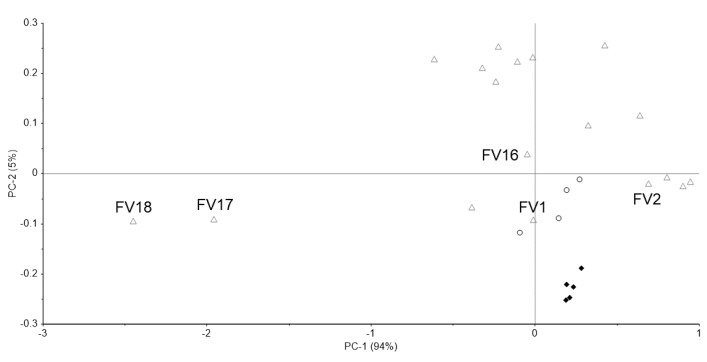
The PCA score plot of the Raman spectra of Viagra tablets. Black diamond; standard tablet (n = 5), black open circle; genuine tablets (n = 4), gray open triangle; falsified tablets (n = 18).

**Figure 11:**
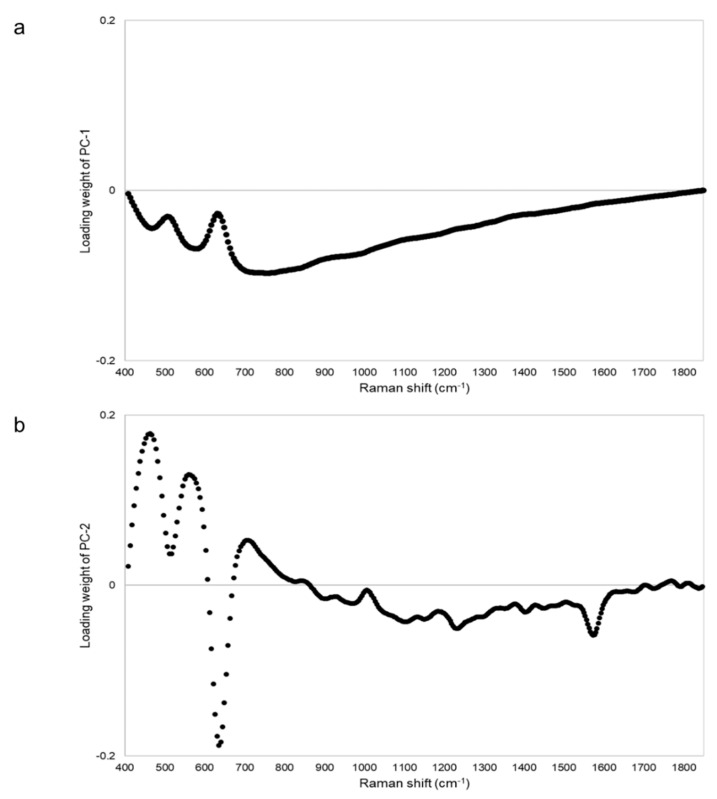
The PCA loading plot of the Raman spectra of Viagra tablets. Panels (**a**) and (**b**) show the loading plots of PC-1 and PC-2 of the Raman spectra of Viagra tablets, respectively.

Figure 10 shows the PCA score plot. PC-1 and PC-2 explained about 99% (PC-1 = 94%, PC-2 = 5%) of the spectra. Genuine tablets (GV1–GV4) were close to the standard tablet. Two falsified tablets (FV17, FV18) with no major peak in Figure 9a were different from the standard tablet in PC-1. The other falsified tablets (FV1–FV16) existed around the standard and genuine tablets in PC-1 and/or PC-2. One falsified tablet (FV1) had almost the same Raman spectra as the other falsified tablets (FV2–FV15), as shown in Figure 9a, but the PCA score plot of FV1 was as close to the standard tablet as the genuine tablets. The score range of PC-2 was about 0.6, indicating minimal differences. Therefore, it might be difficult to completely distinguish between falsified tablets with PCA. PC-1 was affected by the intensity of the peaks around 710–790 cm^−1^ because the absolute value of the loading weight of PC-1 was high around 710–790 cm^−1^ (Figure 11a). PC-2 was affected by the intensity of the peak around 460, 560, and 640 cm^−1^ (Figure 11b).

## 4. Discussion

We have attempted to identify falsified medicines using an ultra-compact Raman spectrometer. All falsified Cialis tablets could be identified by Raman spectra and PCA (Figure 3 and Figure 4). It was possible to identify falsified Levitra tablets by the spectra; however, one of the falsified tablets was close to the standard tablet in the PCA score plot (Figure 6 and Figure 7). Most of the falsified Viagra tablets showed peaks similar to those of the standard tablet (Figure 9a). Falsified tablets did not overlap with the standard and genuine tablets in the PCA score plot, but it was difficult to distinguish all the falsified tablets from the standard tablet even using PCA (Figure 10). The tablets with no major peaks because of fluorescence were all falsified tablets (Figure 3, Figure 6 and Figure 9), indicating that it might be possible to identify falsified tablets by analyzing the Raman spectra as a first step. Furthermore, it was shown that the standard and falsified tablets were different, and there were also differences among falsified tablets, especially in the cases of Cialis and Levitra tablets (Figure 3, Figure 4, Figure 6 and Figure 7). A previous study using a different Raman spectrometer also reported that the Raman spectra of falsified products did not belong to one group [7,8]. We confirmed that the various types of falsified tablets were distributed in the market using an ultra-compact Raman spectrometer.

Standard Cialis, Levitra, and Viagra tablets had peaks at the same wavenumbers, specifically around 510 and 630 cm^−1^. Cialis, Levitra, and Viagra tablets are all film-coated tablets and contain titanium oxide as a coating agent [53,54,56]. These peaks around 510 and 630 cm^−1^ might come from titanium oxide (Figure 2) [30,58,59,60]. The spectra were significantly affected by titanium oxide because the titanium oxide peaks had high intensities compared with the other additives around 510 and 630 cm^−1^. Because the spectral intensity of titanium oxide increases in proportion to the concentration, some falsified tablets with low intensity might contain less titanium oxide than the standard tablet. Titanium oxide is used as an additive mainly for coloring and light blocking; therefore, some falsified tablets might have poor light stability. In this study, the effect of titanium oxide on the Raman spectra was substantial, and the presence of other additives and APIs were not clearly observed in most cases (Figure 2). [61,62,63]. It was suggested that the falsified tablets with different spectra from those of the standard tablet had a different composition of coating agents. Moreover, sildenafil was detected in FL1 and FL2 (Figure 6). Because FL1 and FL2 are falsified tablets containing sildenafil, sildenafil was likely detected due to the failure of the coating layer or the mixing into the coating layer. Most falsified Viagra tablets showed small differences compared with the standard tablet, suggesting that the tablet surface of the falsified tablets was composed of similar ingredients as the standard tablet (Figure 9 and Figure 10). The loading weight of PC-1 and PC-2 for the Raman spectra was weak around 510 and 630 cm^−1^ in most cases (Figure 5a,b, Figure 8a,b and Figure 11a,b). PCA generates a new variable termed the PC that best represents the overall variation. The loading score of the PCA was affected by other peaks, even though the spectra appeared to be affected by titanium oxide.

Some falsified tablets were so similar to the standard tablet that we could not detect all falsified tablets. There were several possible factors. Viagra tablets are supplied as a blue and film-coated tablet. The intensity of blue light is at its maximum at 450 nm and becomes weaker as the wavelength becomes longer. As the excitation wavelength of the ultra-compact Raman spectrometer is 785 nm, the Raman scattered light of Viagra tablets might be difficult to detect because of the blue coating [64,65]. It is important to select an appropriate wavelength. In addition, Raman scattering analysis might be difficult when the surface of the tablet is uneven or curved. Furthermore, in the PCA of Cialis and Viagra tablets, the plots of standard and genuine tablets were slightly far apart and did not overlap (Figure 4 and Figure 10). Differences among the standard and genuine tablets might be affected by the manufacturing country, plant, or process. 

As a result of this study, from the viewpoint of the discrimination of falsified medicines, there was a false negative falsified tablet that could not be distinguished from the standard product even though it was a falsified tablet, suggesting a limitation in the detection of falsified tablets using the ultra-compact Raman spectrometer. However, the tablets with completely different spectra from the standard tablet were falsified products. Therefore, there was no false positive, and rapid non-destructive analysis using Raman spectroscopy allows the number of medicines sent to the laboratory for testing to be reduced. One of the significant advantages of this ultra-compact Raman spectrometer is that it can be introduced in many settings because of its low cost. If the ultra-compact Raman spectrometer was used in medical institutions and customs, it could prevent patients from obtaining falsified medicines and suffering health hazards. The ultra-compact Raman spectrometer also helps to prevent the spread of falsified products in resource-limited settings and LMICs. Although the focus of this study was tablet medicines, the ultra-compact Raman spectrometer is equipped with surface enhanced Raman scattering (SERS), and thus it may also be useful for analyzing liquid samples, such as injections. The emergence of more sophisticated falsified products and the development of detection methods have occurred simultaneously for many years. Although disclosing information regarding the standard product might help with duplicating products, it is considered that publicizing these technological advances and efforts would lead to deterrence in the production of falsified products. The existence of a free spectra library might be useful in developing technologies to detect falsified medicines. We consider that continuing further validation with various types of medicines is important for the implementation of this technique at a large scale.

The products analyzed in this study were limited to medicines for ED purchased over the internet in previous studies. There may be many kinds of falsified medicines being marketed around the world. Validation experiments with other types of medicines are required. Because the number of tablets in this study was limited, data were only obtained from one tablet in each case; given that falsified medicines with poor quality do not have the same properties among packages or batches, this approach might require measuring multiple tablets per product.

## 5. Conclusions

An ultra-compact Raman spectrometer was used to detect falsified medicines for ED. Although successful for Cialis and Levitra, there was only limited success at identifying some falsified Viagra tablets. Therefore, the limitation of identification should be considered. Detecting falsified medicines in the field using a portable analyzer could help to prevent the spread of falsified medicine and protect the health of patients. Low-cost analyzers could be used in various settings, such as LMICs, medical institutions, and customs.

## Data Availability

The data presented in this study are available on request from the corresponding author. The data are not publicly available due to avoid helping to manufacture falsified products.
